# Polymorphism in ERCC1 confers susceptibility of coronary artery disease and severity of coronary artery atherosclerosis in a Chinese Han population

**DOI:** 10.1038/s41598-017-06732-9

**Published:** 2017-07-25

**Authors:** Shuai Zhang, Xue-bin Wang, Ya-di Han, Chen-ling Xiong, Ye Zhou, Chen Wang, Ze-jin Liu, Na Yang, Fang Zheng

**Affiliations:** 1 Center for Gene Diagnosis, Zhongnan Hospital of Wuhan University, Wuhan, Hubei China; 2 Center of Clinical Laboratory, Wuhan Asia Heart Hospital, Wuhan, Hubei China

## Abstract

Excision repair cross-complementing 1 (ERCC1) gene encodes ERCC1 protein, which is mainly responsible for the repair of DNA damage in different diseases including coronary artery atherosclerosis by acting as a rate-limiting element in nucleotide excision repair (NER). Using a three-stage case-control study with 3037 coronary artery disease (CAD) patients and 3002 controls, we investigated associations of three single nucleotide polymorphisms (SNPs) with CAD risk and severity of coronary artery atherosclerosis in Chinese Han population. In the discovery set, the variant allele T of rs11615 was significantly associated with higher CAD risk (adjusted OR = 1.27, P = 0.006) and severity of coronary artery atherosclerosis (adjusted OR = 1.54, P = 0.003). These associations were more remarkable in the merged set (adjusted OR = 1.23, P = 8 × 10^−6^ for CAD risk; adjusted OR = 1.36, P = 4.3 × 10^−5^ for severity of coronary artery atherosclerosis). And the expression level of ERCC1 was significantly higher in CAD cases than controls. Multiplicative interactions among SNP rs11615, alcohol drinking, history of T2DM, and history of hyperlipidemia could increase 5.06-fold risk of CAD (P = 1.59 × 10^−9^). No significant association of rs2298881 and rs3212986 with CAD risk was identified. Taken together, SNP rs11615 in ERCC1 gene might confer susceptibility to CAD and severity of coronary atherosclerosis in a Chinese Han population.

## Introduction

Coronary artery disease (CAD) is the most common heart disease, which is caused by stenosis in coronary arteries and its branches due to genetic and environmental factors^1^. The main pathogenesis of CAD is atherosclerosis, a form of chronic inflammation characterized by accumulated deposition of lipoproteins, migration of monocyte and macrophage, and eventually atheroma formation and rupture^2, 3^. Recent publications report that CAD is etiologically related to DNA damages caused by reactive oxygen species (ROS)^4^, lipoprotein oxidation and peroxidation^5^. DNA damage associating with atherosclerosis occurs in circulating cells, vascular smooth muscle cells (VSMCs) and endothelial cells of the vessel wall. Unrepaired DNA damage promotes endothelial cell dysfunction, monocyte migration, macrophage and foam cell death, VSMC senescence, and eventually contributes to plague rupture via inducing expression of adhesion molecules and release of inflammatory cytokines^6–8^. One of the most important DNA repair systems is nucleotide excision repair (NER), which is highly versatile and involved in repairing a wide variety of sophisticated DNA damage, such as helix-distorting DNA lesions and DNA adducts, induced by endogenous and exogenous stimuli^9, 10^.

Excision repair cross-complementing 1 (ERCC1) is the rate-limiting element in NER system^11^. ERCC1 is mainly responsible for making incision at the 5′ terminus of DNA lesions by forming a structure-specific endonuclease complex with xeroderma pigmentosum complementation group F (XPF) and may contribute to positioning the nuclease complex at the lesion site by interacting with Xeroderma pigmentosum complementation group A (XPA)^9^. Besides evidence of molecular studies, in animal model studies, the knockdown and overexpression of ERCC1 was related highly to the increase and decrease of infarct volume in the ischemic brain respectively^12^. Mice with ERCC1 deficiency showed vascular dysfunctions, including elevated blood pressure and increased vascular stiffness^13^ and also led to elevated serum cholesterol level and the expression change of genes involved in metabolism of cholesterol, which have been considered as traditional risk factors for CAD^14, 15^. Based on the function of ERCC1 and the effect of ERCC1 deficiency on brain ischemia, vascular dysfunction, and cholesterol metabolism, we guessed that the SNPs related to ERCC1 expression might also contribute to CAD risk since CAD and stroke are both belong to atherosclerosis cardiovascular diseases due to high cholesterol levels. Then, we chose three SNPs in ERCC1 for further study. One is synonymous C19007T (rs11615) polymorphism locating at exon IV, which has been reported to be associated with differential ERCC1 mRNA and protein levels^16^. The other is C8092A (rs3212986) in 3′-untranslated region (UTR) and may regulate ERCC1 expression by affecting ERCC1 mRNA stability^17^. The last is rs2298881 locating in intron I, which has been reported to alter ERCC1 expression by affecting ERCC1 promoter activity^18^.

Therefore, in the present study, we performed a three-stage case-control study to investigate the association of these three SNPs with CAD risk and severity of coronary artery atherosclerosis in a Chinese Han population.

## Results

### Clinical characteristics of study population

Baseline and clinical characteristics of study populations were shown in Table 1. Statistically significant differences between cases and controls in three independent populations of our study were found in body mass index (BMI) and the frequencies of smoking, alcohol drinking, hypertension, T2DM and hyperlipidemia. The genotype distribution of all three SNPs in controls was consistent with Hardy-Weinberg equilibrium (HWE) (Supplementary Table S1).

**Table 1:** Baseline and clinical characteristics of study populations.

Variables	Discovery set (Study 1)			Validation set (Study 2)			Replication set (Study 3)		
	CAD (N = 806)	Control (N = 816)	P*	CAD (N = 1124)	Control (N = 1118)	P*	CAD (N = 1107)	Control (N = 1068)	P*
Age, years	62.5 ± 9.6	62.4 ± 10.7	0.804	62.5 ± 9.6	62.5 ± 10.9	0.708	62.2 ± 9.7	62.3 ± 11.9	0.655
Male, n (%)	482 (59.8)	490 (60.0)	0.919	669 (59.5)	674 (60.3)	0.711	698 (63.1)	688 (64.4)	0.508
BMI, kg/m^2^	25.1 ± 4.1	24.1 ± 2.2	<0.001	25.1 ± 3.8	24.1 ± 2.2	<0.001	25.3 ± 8.6	23.3 ± 6.8	<0.001
Smoking, n (%)	284 (35.2)	234 (28.7)	0.005	472 (42.0)	301 (26.9)	<0.001	491 (44.4)	313 (29.3)	<0.001
Alcohol drinking, n (%)	280 (34.7)	208 (25.5)	<0.001	325 (28.9)	268 (24.0)	0.008	389 (35.1)	316 (29.6)	0.006
Hypertension, n (%)	472 (58.6)	346 (42.4)	<0.001	653 (58.1)	402 (36.0)	<0.001	684 (61.8)	448 (41.9)	<0.001
T2DM, n (%)	246 (30.5)	212 (26.0)	0.042	448 (39.9)	299 (26.7)	<0.001	437 (39.5)	343 (32.1)	<0.001
Hyperlipidemia, n (%)	245 (30.4)	184 (22.5)	<0.001	419 (37.3)	260 (23.3)	<0.001	419 (37.9)	249 (23.3)	<0.001
Gensini score	32 (21.5–77.8)	—		32 (15–66.5)	—		33 (17.5–66.5)	—	

*For continuous variables, normally distributed data are expressed as mean ± standard deviation (SD), while skewed data are described as median (interquartile range). For categorical, data are expressed as frequency counts.

### Association results of ERCC1 SNPs with CAD risk

In the discovery set, significantly increased CAD risk was found with the minor allele T of SNP rs11615 (adjusted OR = 1.27, P = 0.006) after adjusting for age, sex, BMI, smoking, alcohol drinking, hypertension, T2DM and hyperlipidemia (Supplementary Table S2). For further verifying accuracy of this significant association, we genotyped SNP rs11615 in the following two sets and obtained similar significant results (adjusted OR = 1.19, P = 0.021 in validation set and adjusted OR = 1.23, P = 0.01 in replication set) (Supplementary Table S2). Moreover, this association became much more remarkable in the combined set (3037 cases and 3002 controls) with an adjusted OR of 1.23 and an adjusted P value of 8 × 10^−6^ (Table 2). Assuming a minor allele frequency (MAF) of 0.258 and 0.222 in CAD and control respectively and OR of 1.23, the combined set could provide a power of 92.4% to detect the association with the type I error of 0.05.

**Table 2:** Association of ERCC1 SNPs with CAD risk in 3037 CAD patients and 3002 controls.

SNPs	Alleles/genotypes N (%)		OR (95% CI)*	P*/P_BON_^†^
	CAD	Control		
rs11615				
C	4506 (74.2)	4673 (77.8)	1 (Ref)	
T	1568 (25.8)	1331 (22.2)	**1**.**23** (**1**.**12**–**1**.**34**)	<**0**.**001/**<**0**.**001**
CC	1666 (54.9)	1802 (60.0)	1 (Ref)	
CT	1174 (38.7)	1069 (35.6)	**1**.**19** (**1**.**06**–**1**.**34**)	**0**.**003/0**.**009**
TT	197 (6.5)	131 (4.4)	**1**.**65** (**1**.**29**–**2**.**11**)	<**0**.**001/**<**0**.**001**
TT + CT	1351 (44.5)	1200 (40.0)	**1**.**24** (**1**.**11**–**1**.**39**)	<**0**.**001/**<**0**.**001**
rs2298881				
C	2411 (62.5)	2384 (61.6)	1 (Ref)	
A	1449 (37.5)	1484 (38.4)	0.96 (0.87–1.06)	0.40
CC	753 (39.0)	742 (38.4)	1 (Ref)	
AC	905 (46.9)	900 (46.5)	0.96 (0.83–1.11)	0.536
AA	272 (14.1)	292 (15.1)	0.92 (0.75–1.13)	0.444
AA + AC	1133 (61.0)	1192 (61.6)	0.95 (0.83–1.09)	0.44
rs3212986				
G	2647 (68.6)	2595 (67.1)	1 (Ref)	
T	1213 (31.4)	1273 (32.9)	0.94 (0.85–1.04)	0.248
GG	910 (47.2)	872 (45.1)	1 (Ref)	
GT	827 (42.8)	851 (44.0)	0.96 (0.83–1.10)	0.523
TT	193 (10.0)	211 (10.9)	0.88 (0.70–1.10)	0.253
TT + GT	1020 (52.8)	1062 (54.9)	0.94 (0.82–1.07)	0.358

CAD, coronary artery disease; N: number; OR (95% CI): odds ratio (95% confidence interval); Ref: reference. *Adjusted OR (95% CI) and P values were obtained from logistic regression analyses after adjusting for age, sex, BMI, smoking status, alcohol drinking and histories of T2DM, hyperlipidemia and hypertension. ^†^Multiple testing by the Bonferroni correction, P-value multiplied 3 (3 SNPs) to get a P_BON_ value. Bold values are statistically significant with P < 0.05.

We then used the dominant model to perform genotypic analyses for exploring its potential effect on CAD risk. In the discovery set, we identified an important association of SNP rs11615 with CAD risk in the dominant model (adjusted OR = 1.27, P = 0.022) (Supplementary Table S2). this significant association, having been verified in validation set and replication set, remained unchanged in the combined set (adjusted OR = 1.24, P = 1 × 10^−4^ in the dominant model) (Table 2). All significant associations in the combined set continued to be meaningful after Bonferroni correction (Table 2).

However, we found no evidence supporting the association of SNP rs2298881 and rs3212986 with CAD risk in Chinese (Table 2 and Supplementary Table S2).

### Subgroup analyses on the association of SNP rs11615 with CAD risk

Using a dominant model with and without adjustment for covariates, subgroup analyses found significant associations of variant genotypes (CT + TT) of SNP rs11615 with increased CAD risk in almost all subgroups, expect for participants without alcohol drinking habit (Table 3). After the Bonferroni correction, significant associations also remained in the elder subgroup (>60) and participants with alcohol drinking, T2DM and hyperlipidemia (Table 3). Besides, multiplicative likelihood ratio test indicated that variant genotypes (CT + TT) of SNP rs11615 interacted with alcohol drinking (P_inter_ = 0.004), T2DM (P_inter_ = 0.013) and hyperlipidemia (P_inter_ = 0.018) to increase CAD risk (Table 3).

**Table 3:** Subgroup analyses for the association of SNP rs11615 with CAD risk.

Variables	SNP rs11615 (cases/controls, N)		Without adjustment		With adjustment*		P_inter_^†^
	CC	CT + TT	OR (95% CI)	P	OR (95% CI)	P_adj_	
Age, years							
≤60	797/882	600/572	1.16 (1.00–1.35)	0.05	1.20 (1.02–1.41)	0.027	0.280
>60	869/920	771/628	**1**.**30** (**1**.**13**–**1**.**50**)	<**0**.**001**	**1**.**28** (**1**.**10**–**1**.**49**)	**0**.**001**	
Sex							
Male	1031/1121	818/731	1.22 (1.07–1.39)	0.003	1.22 (1.06–1.41)	0.007	0.718
Female	635/681	553/469	1.27 (1.07–1.49)	0.005	1.25 (1.05–1.49)	0.013	
BMI, kg/m^2^							
≤25	877/1199	696/799	1.19 (1.04–1.36)	0.011	1.19 (1.03–1.36)	0.016	0.475
>25	751/561	638/370	1.29 (1.09–1.52)	0.003	1.30 (1.08–1.56)	0.006	
Smoking status							
Yes	675/513	572/335	1.30 (1.09–1.55)	0.004	1.26 (1.04–1.52)	0.018	0.481
No	991/1289	798/865	1.20 (1.06–1.36)	0.005	1.19 (1.03–1.36)	0.015	
Alcohol drinking							
Yes	522/502	472/290	**1**.**57** (**1**.**29**–**1**.**89**)	<**0**.**001**	**1**.**43** (**1**.**16**–**1**.**76**)	**0**.**001**	0.004
No	1144/1300	899/910	1.12 (0.99–1.27)	0.062	1.15 (1.01–1.31)	0.042	
Hypertension							
Yes	1008/733	801/463	1.26 (1.08–1.46)	0.002	1.22 (1.04–1.42)	0.015	0.991
No	658/1069	570/737	1.26 (1.09–1.45)	0.002	1.28 (1.09–1.49)	0.002	
T2DM							
Yes	608/543	523/311	**1**.**50** (**1**.**25**–**1**.**80**)	<**0**.**001**	1.33 (1.09–1.62)	0.005	0.013
No	1053/1259	846/889	1.14 (1.01–1.29)	0.043	1.19 (1.04–1.36)	0.029	
Hyperlipidemia							
Yes	572/435	511/258	**1**.**51** (**1**.**24**–**1**.**83**)	<**0**.**001**	**1**.**45** (**1**.**17**–**1**.**80**)	**0**.**001**	0.018
No	1094/1367	860/942	1.14 (1.01–1.29)	0.034	1.16 (1.02–1.32)	0.027	

N, number; OR (95CI), odds ratio (95% confidence interval); SNP, single nucleotide polymorphism; BMI, body mass index; T2DM, type 2 diabetes mellitus. *Adjusted OR (95% CI) and P_adj_ values were obtained from logistic regression analyses after adjusting for age, sex, BMI, smoking status, alcohol drinking and histories of T2DM, hyperlipidemia and hypertension. ^†^P_inter_ values were obtained from the multiplicative likelihood ratio test to assess the interactions between SNP rs11615 and selected variables in CAD risk. Bold values indicate statistically significant after the Bonferroni correction (P < 0.05/40 = 0.00125).

### Classification and regression tree (CART) analyses

**Figure 1:**
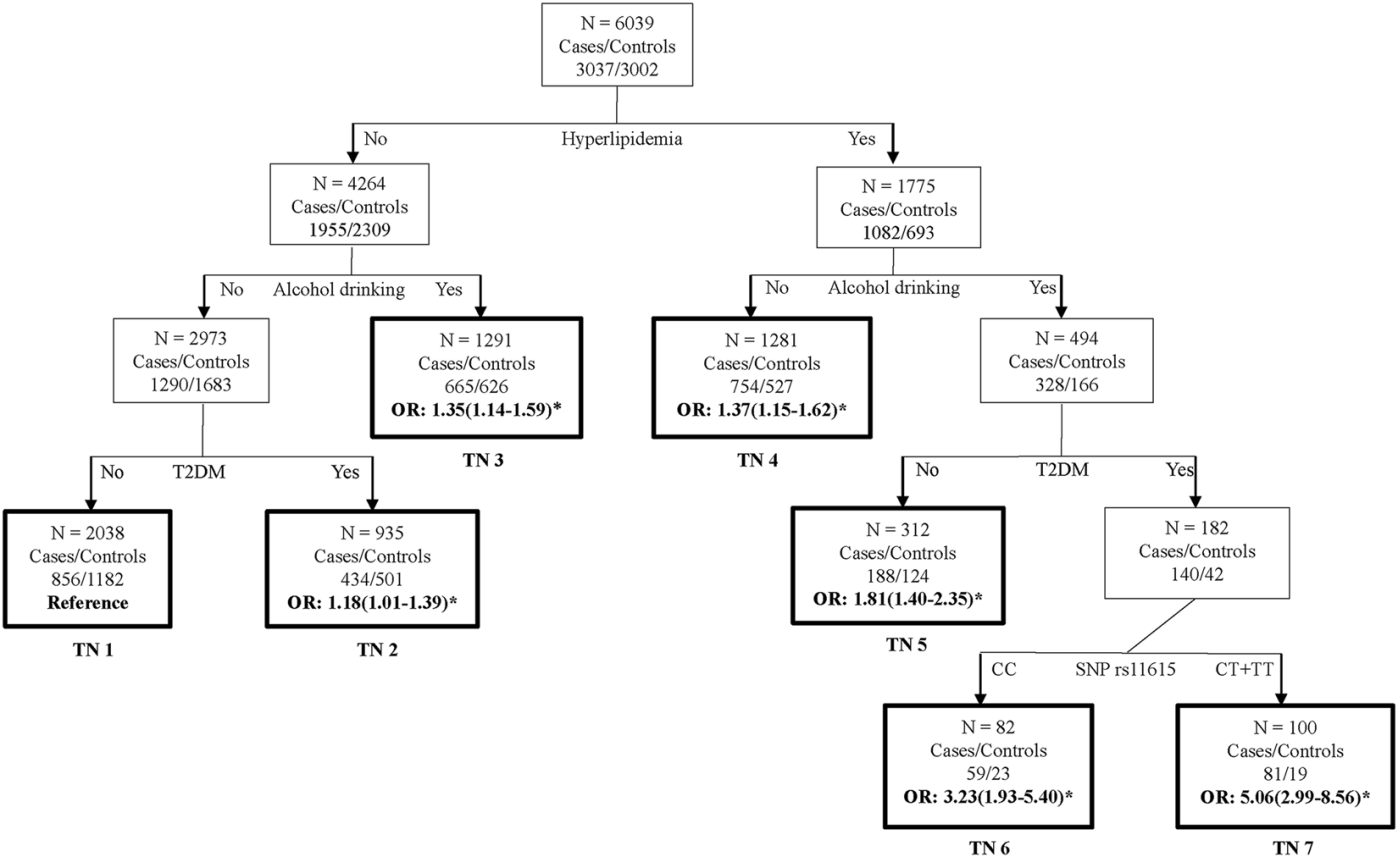
Classification and regression trees for alcohol drinking, history of T2DM and hyperlipidemia and SNP rs11615 in all participants of our study. Terminal nodes (TN) are thick bordered. ORs and 95% CIs were calculated by logistic regression after adjusting for age, sex, BMI, smoking, alcohol drinking and histories of hypertension, hyperlipidemia and T2DM, *P < 0.05.

Alcohol drinking, T2DM, hyperlipidemia and SNP rs11615 information were included in classification and regression tree (CART) analyses based on the result of multiplicative interaction analyses. Hyperlipidemia had the greatest influence on CAD risk among the factors included, which consisted with it being the initial split node in CART analyses. Subsequent inspection of the CART tree revealed a 5.06-fold (P = 1.59 × 10^−9^) increased risk of CAD in the participants with variant genotypes (CT + TT) of SNP rs11615, alcohol drinking and history of T2DM and hyperlipidemia compared to the reference group, which may be an evidence of genetic and environmental interactions (Fig. 1).

### Associations of SNP rs11615 with severity of coronary artery atherosclerosis

**Figure 2:**
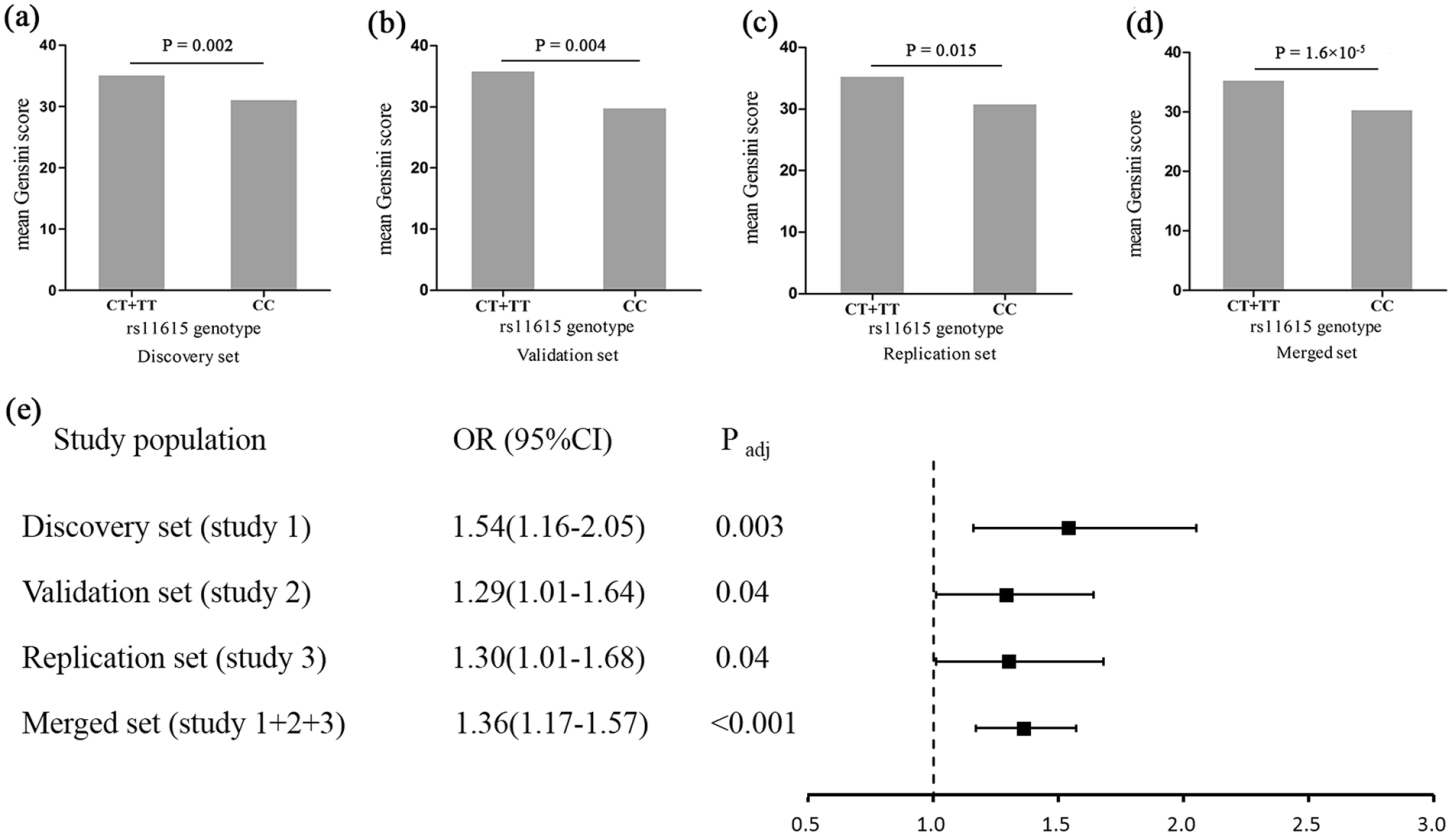
Genetic estimates of association of SNP rs11615 with Gensini score. Variant genotypes (CT + TT) of SNP rs11615 was associated with higher Gensini score (median) in the discovery set (**a**), validation set (**b**), replication set (**c**) and the merged set (**d**) and also associated with increased risk of severity of coronary artery atherosclerosis in independent or merged set (**e**). P, non-adjusted P; P_adj_, adjusted P.

For SNP rs11615, Gensini scores increased obviously from the CC carriers to the CT + TT carriers in the discovery set (P = 0.002), validation set (P = 0.004), replication set (P = 0.015) and merged set (P = 1.6 × 10^−5^) (Fig. 2). Then, we classified CAD cases into two groups based on the median of Gensini scores (32.5). Multivariate logistic regression analyses indicated that the variant genotypes (CT + TT) of SNP rs11615 were associated with higher Gensini scores in the merged set (adjusted OR = 1.36, P = 4.3 × 10^−5^) (Fig. 2). These associations were more distinct in female and hypertension subgroups and still statistically significant after Bonferroni correction (Table 4).

**Table 4:** Association of SNP rs11615 with the severity of coronary atherosclerosis.

Variables	Gensini scores*					Gensini scores^‡^ (≤32.5/>32.5)		With adjustment^‡^		P_inter_^§^
	N	CC	N	CT + TT	P^†^	CC	CT + TT	OR (95% CI)	P_adj_	
Age, years										
≤60	797	29.5 (15.5–68.0)	600	33.0 (20.5–65.5)	0.004	443/354	287/313	1.34 (1.08–1.67)	0.008	0.90
>60	869	31.5 (16.0–71.3)	771	37.5 (20.5–71.5)	0.002	457/412	344/427	1.37 (1.12–1.67)	0.002	
Sex										
Male	1031	32.5 (17.0–74.5)	818	36.5 (21.5–70.8)	0.037	516/515	360/458	1.25 (1.03–1.51)	0.022	0.14
Female	**635**	**26**.**0** (**14**.**5**–**61**.**0**)	**553**	**33**.**0** (**19**.**0**–**65**.**5**)	<**0**.**001**	384/251	271/282	**1**.**57** (**1**.**24**–**1**.**99**)	<**0**.**001**	
BMI, kg/m^2^										
≤25	877	29.0 (16.0–67.3)	696	33.0 (20.5–66.3)	0.002	494/383	339/357	1.35 (1.10–1.65)	0.004	0.72
>25	751	32.0 (15.5–72.0)	638	38.3 (20.5–71.5)	0.009	388/363	280/358	1.36 (1.10–1.68)	0.005	
Smoking status										
Yes	675	32.0 (17.0–71.5)	576	37.0 (21.5–68.5)	0.041	347/328	249/323	1.37 (1.09–1.73)	0.007	0.86
No	991	29.0 (15.0–70.0)	798	33.5 (19.4–70.1)	0.003	553/438	382/416	1.36 (1.13–1.65)	0.002	
Alcohol drinking										
Yes	522	32.0 (16.9–74.1)	472	35.3 (21.0–66.9)	0.098	267/255	209/263	1.28 (0.99–1.65)	0.058	0.46
No	1144	29.5 (15.5–68.0)	899	35.0 (20.5–71.5)	0.003	633/511	422/417	1.42 (1.19–1.71)	0.003	
Hypertension										
Yes	**1008**	**29**.**0** (**15**.**1**–**68**.**9**)	**801**	**37**.**0** (**20**.**5**–**71**.**5**)	<**0**.**001**	566/442	361/440	**1**.**55** (**1**.**28**–**1**.**87**)	<**0**.**001**	0.04
No	658	32.0 (16.9–72.1)	570	33.5 (20.0–65.1)	0.146	334/324	270/300	1.14 (0.90–1.44)	0.272	
T2DM										
Yes	608	36.0 (18.5–72.9)	523	41.5 (21.5–73.0)	0.038	294/314	213/310	1.36 (1.07–1.74)	0.012	0.94
No	1053	29.0 (14.5–68.3)	846	33.0 (19.9–66.5)	0.001	602/451	416/430	1.36 (1.13–1.64)	0.002	
Hyperlipidemia										
Yes	572	29.0 (15.5–66.5)	511	36.0 (20.5–65.5)	0.003	315/257	234/277	1.44 (1.13–1.85)	0.004	0.62
No	1094	31.0 (16.0–72.5)	860	35.5 (20.5–70.4)	0.002	585/509	397/463	1.32 (1.10–1.59)	0.003	

N, number; OR (95CI), odds ratio (95% confidence interval); BMI, body mass index; T2DM, type 2 diabetes mellitus. *Gensini scores are expressed as median (interquartile range) because of the skewed distributions. ^†^P values were obtained from the Mann-Whitney U test. ^‡^CAD patients were classified into two groups based on the median (32.5) of Gensini scores, and then adjusted OR (95% CI) and P_adj_ values were obtained from logistic regression analyses after adjusting for age, sex, BMI, smoking status, alcohol drinking and histories of T2DM, hyperlipidemia and hypertension. ^§^P_inter_ values were obtained from the multiplicative likelihood ratio test to assess the interactions between SNP rs11615 and selected variables in CAD risk. Bold values indicate statistically significant after the Bonferroni correction (P < 0.05/48 ≈ 1.04 × 10^−3^).

### Correlations of SNP rs11615 with ERCC1 mRNA expression and plasma ERCC1 level

**Figure 3:**
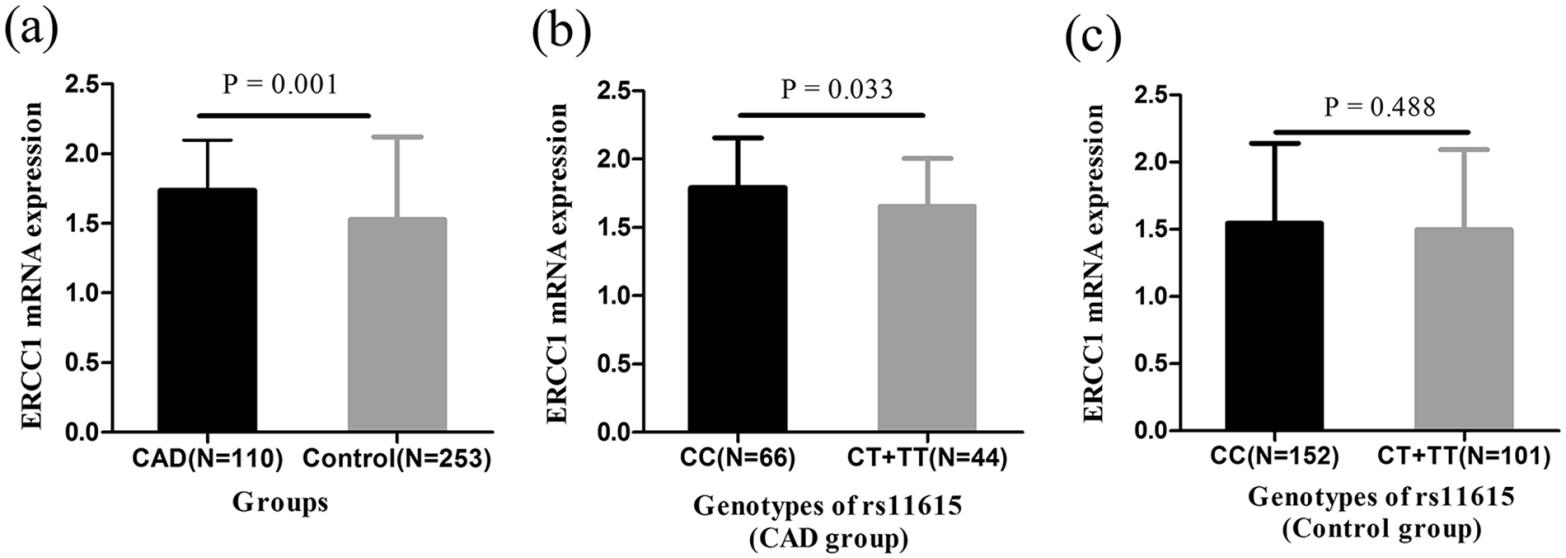
Associations of SNP rs11615 with ERCC1 mRNA expression. ERCC1 mRNA expression (1.74 ± 0.36 vs 1.53 ± 0.59) was higher in CAD group than control group (**a**); Associations of SNP rs11615 with ERCC1 mRNA expression in CAD group (1.79 ± 0.36 vs 1.66 ± 0.35) (**b**) and control group (1.55 ± 0.59 vs 1.50 ± 0.60) (**c**). ANCOVA models are used to assess statistical significance. Data are expressed as mean ± standard deviation (SD).

We measured ERCC1 mRNA expression in 363 subjects (110 CAD patients vs 253 controls) and plasma ERCC1 level in 78 subjects (39 CAD patients vs 39 controls), all of whom were randomly selected from the validation and replication sets. After adjusting for covariates, ANCOVA models showed that ERCC1 mRNA expression (1.74 ± 0.36 vs 1.53 ± 0.59, P = 0.001) were higher in CAD patients than controls and then a significant association was found between variant genotypes (CT + TT) and decreased ERCC1 mRNA expression in CAD patients (P = 0.033) but not in controls (P = 0.488) (Fig. 3). Moreover, CAD patients had a higher plasma ERCC1 level than controls (612.35 ± 82.33 vs 580.62 ± 65.85, P = 0.239), but without reaching statistically difference. No association was discovered between SNP rs11615 genotypes and plasma ERCC1 level.

## Discussion

This three-stage case-control study with 3037 CAD cases and 3002 controls, for the first time, revealed that the minor allele T of SNP rs11615 was associated with significantly increased CAD risk in Chinese Han population, especially in the elder subgroup (>60) and participants with alcohol drinking, T2DM and hyperlipidemia. And CART analyses shown that variant genotypes (CT + TT) of SNP rs11615, combined with the above three CAD risk factors, could increase CAD risk up to 5.06 times. Furthermore, variant genotypes (CT+TT) of SNP rs11615 contributed to the severity of coronary artery atherosclerosis. In addition, it presented that CAD patients had a significantly higher ERCC1 mRNA expression levels than controls, however we found the statistical association between the CT + TT genotype and a lower ERCC1 expression level only in CAD patients.

Actually previous studies have reported that mice with ERCC1 deficiency showed a higher mutation frequency, increased genomic instability, the dramatic accumulation of unrepaired lesions and then the development of cardiovascular disease like CAD^13, 19^. In this study, we found a higher frequency of variant genotypes (CT + TT) in CAD patients. And the patients with variant genotypes (CT + TT) had a decreased level of ERCC1 expression. Besides, a previous study in cell lines got coincident results that ERCC1 levels in human ovarian cancer cell (MCAS) with T allele of SNP rs11615 reduced almost 60% of that in human ovarian cancer cell (A2781/CP70) with wild-type ERCC1 sequence^20^. So we inferred that the T allele of SNP rs11615 was associated with increased risk and severity of CAD in Chinese Han population, probably by reducing the expression level of ERCC1 compared to the C allele. However, potential mechanism needs to be further studied.

In regard to the mechanism under the association between rs11615 genotype and ERCC1 expression, it is widely accepted that the synonymous polymorphism (rs11615) at codon 118 of the ERCC1 gene, converting a high-usage codon (AAC) to a relatively infrequent one (AAT), could affect ERCC1 translation and the level of ERCC1 protein and thereby impair repair activity on account of codon usage being decreased by almost half^21, 22^. Subsequent bioinformatics using ENCODE at UCSC (http://genome.ucsc.edu/ENCODE/) further revealed that variant allele of SNP rs11615 changed the binding site of ZNF263 transcription factors, which might have the ability to influence the expression of the target gene^23^. Moreover, Epigenome Browser (http://epigenomegateway.wustl.edu/) also showed that the wild type of SNP rs11615 located in a region having potential enhancer activity and the variant allele of this locus had the possibility to relatively down-regulate ERCC1 expression (Supplementary Fig. S1). Therefore, we speculated that SNP rs11615 had the ability to increase CAD risk by decreasing ERCC1 expression levels in the transcription level instead of translation level.

In the present study, we just found the association of variant genotypes of SNP rs11615 with ERCC1 mRNA expression levels in CAD patients, but not in controls. These results could arise from the restriction of small sample study, or dominants of DNA damage in patients, or because peripheral blood cells are not in step with plaque tissue. DNA damage occurred mainly in atherosclerotic plaque, ranging from deletions of parts of chromosomes to DNA strand breaks and DNA adducts and that was more serious than DNA damage in peripheral white blood cells^7, 24^. It is common knowledge that differential extent of DNA damage accompanied with varying degree of expression of DNA repair-related genes. Consistent with this, immunoreactivity for 7,8-dihydro-8-oxo-2′-deoxyguanosine (8-oxo-dG), an oxidative DNA damage marker, was detected in atherosclerotic plaque VSMCs, macrophages and endothelial cells, but not in VSMCs of adjacent normal media^25^. Therefore, ERCC1 expression levels in peripheral blood, only relatively reflect the repair situation of coronary artery in CAD patients to a certain extent, and future studies are needed to detect ERCC1 expression in atherosclerotic plaque.

To explore potential genetic and environmental interaction effect on CAD risk, we performed CART analyses and found that participants with variant genotypes (CT + TT) of SNP rs11615, alcohol drinking and history of T2DM and hyperlipidemia tended to have increased CAD risk. Heavy alcohol drinking always generated a great deal of reactive oxygen species (ROS), which subsequently induced a variety of DNA damage as well as low density lipoprotein (LDL) oxidation^26^. Accumulation of the unrepaired DNA damage which resulted directly from alcohol drinking or indirectly from DNA repair enzyme (ERCC1) deficiency, contributed to the progression of CAD by activating the release of inflammatory cytokines^13^. And it was reported that interaction of SNP rs11615 with alcohol drinking could increase the risk of laryngeal cancer^27^. For history of T2DM, recent studies have shown that abnormal glucose metabolism could enhance ERCC1 expression and protein levels by activating the release of insulin and that mice with ERCC1 deficiency showed a progeroid phenotype with disturbance of glucose metabolism^28, 29^. As for hyperlipidemia, lipid oxidation and peroxidation was common reason for DNA damage and then aggravated the development of atherosclerosis. By the way, ERCC1 deficiency could affect lipid metabolism by up-regulating genes related to extracellular efflux of cholesterol^14, 15^. All the above evidence, combined with the proved effect of SNP rs11615 on ERCC1 expressions, support that development of CAD could be further aggravated by interaction between SNP rs11615 and traditional CAD factors (alcohol drinking, T2DM and hyperlipidemia). However, further functional studies are needed to explore the potential mechanism of this interaction.

The present study evaluated severity of coronary artery atherosclerosis by calculating Gensini score and revealed that variant genotype (CT + TT) of SNP rs11615 was associated significantly with higher Gensini scores. This result was supported by a recent study that knockdown of ERCC1 expression exhibited an increasing infarct volume in the ischemic rat brain^12^ and the report that ERCC1-deficient mice showed increased vascular stiffness and vascular dysfunction^13^. Therefore, combined with the association of SNP rs11615 with ERCC1 expression, we speculated that SNP rs11615 might accelerate the progress of coronary artery atherosclerosis.

Some limitations should be taken into consideration. First, we just genotyped three most common SNPs in ERCC1 and failed to explore the effect of other ERCC1 genetic variants on CAD risk. Second, although we have adjusted for common CAD risk factors, other genetic and environmental factors could also involve the development of CAD. Finally, ERCC1 expression and protein levels in plaques were not detected in our study due to restriction of samples.

In conclusion, this three-stage case-control study of 3037 cases and 3002 controls, for the first time, suggested significant association of SNP rs11615 with CAD risk as well as possible interactions among SNP rs11615, alcohol drinking and history of T2DM and hyperlipidemia. Functional studies are required to validate our findings and illuminate the potential mechanism.

## Materials and Methods

### Study population

This three-stage case-control study, involving 3037 CAD patients and 3002 controls, was selected from three sets: the discovery set (study 1) with 806 CAD cases and 816 controls from Zhongnan Hospital of Wuhan University between January 2011 and December 2012; the validation set (study 2) with 1124 CAD cases and 1118 controls from Wuhan Asia Heart Hospital between March 2013 and October 2014; and the replication set (study 3) with 1107 CAD cases and 1068 controls from the above two centers between March 2015 and May 2016. All study participants were Han nationality by self-description.

The diagnosis criteria of CAD was stenosis of more than 50%, confirmed by coronary angiography, in at least one segment of main coronary artery or their main branches. Patients with the following diseases were excluded: cardiac diseases including acute heart failure, congenital heart disease, myocardial bridge or cardiomyopathy and coronary artery spasm, as well as systemic disease such as malignancy, autoimmune disease, severe liver or renal disease and immunosuppressive drug use. Controls were age- and gender-matched participants without detectible luminal stenosis identified by coronary angiography (1054 controls) and healthy individuals without above-mentioned cardiac or systemic diseases discovered by physical examination (1948 controls). The following data were extracted, traditional CAD risk factors^30, 31^ including cigarette smoking, alcohol drinking and histories of hypertension, type 2 diabetes mellitus (T2DM) and hyperlipidemia (Supplementary materials and methods) and clinical data, such as blood pressure, body mass index (BMI), fasting plasma glucose (FPG) and lipid levels. This study and informed consent were approved by Medical Institutional Review Board of Zhongnan Hospital of Wuhan University and conformed to guidelines of the Declaration of Helsinki.

### Selection of SNPs and genotyping

A large number of studies investigated the association of single nucleotide polymorphisms (SNPs) in ERCC1 gene with cancer. Based on the 1000 Genome Database (http://www.1000genomes.org/)^32^, three most common SNPs, which were rs11615 in exon IV, rs3212986 in 3′-untranslated region (UTR), and rs2298881 in intron I, were selected (Supplementary Table S1).

We extracted genomic DNA from peripheral blood leucocytes by using a phenol/chloroform method and then genotyped 3 SNPs by high-throughput sequencing using illumina Miseq system (Illumina, San Diego, CA) (Supplementary genotyping methods and Supplementary Fig. S2)^33, 34^. Direct PCR sequencing was performed to confirm the accuracy of genotyping (Supplementary Fig. S3). Detailed information for genotyping and direct sequencing, such as primer sequences and PCR conditions, was exhibited in Supplementary Table S3.

### Scoring of coronary angiogram

In the Gensini scoring system^35, 36^, angiographic stenosis of each coronary artery segment was scored as 1 point for the range of 0–25%, 2 for 26–50%, 4 for 51–75%, 8 for 76–90%, 16 for 91–99% and 32 for 100%. Then, each coronary artery branch corresponds to a coefficient, ranging from 0.5 to 5, depending on the location of stenosis and importance of areas supplied by that segment. A patient’s final Gensini score is the sum of the weighted scores for each stenosis segment.

### CART analyses

To evaluate the potential high-order genetic and environmental interactions in CAD risk, we carried out classification and regression tree (CART)^37^ analyses by using Clementine 12.0 (SPSS Inc, Chicago, IL, USA) programs. CART adopted Gini index as splitting criterion to build a hierarchical classification tree for finding an optimal combination of genetic and environmental factors, which could predict CAD risk more forcefully^38^. Logistic regression analyses were used to assess the association of each terminal node (TN) with CAD risk.

### Real-time quantitative PCR analysis of ERCC1 mRNA expression

After extracting from human peripheral blood leukocytes using Trizol reagent (Invitrogen, Carlsbad, CA, USA), total RNA was prepared to remove DNA contamination using the RNase-Free gDNA eraser and then conduct reverse transcription using a reverse transcriptase kit (Takara Bio Inc, Kusatsu, Shiga, Japan). The cDNA product was used to determine ERCC1 mRNA expression using real-time quantitative PCR (RT-qPCR) analysis with the SYBR-Green kit on a CFX96 Touch system (Bio-rad, Hercules, CA, USA). The 2^−ΔΔCq^ method was used to calculate the relative expression of ERCC1 by normalizing to the internal reference gene (GAPDH).

### Measurement of plasma ERCC1 levels

Plasma samples were isolated from the whole blood by centrifugation and stored at −80 °C immediately until use. According to the manufacturer’s instructions, we measured plasma concentration of ERCC1 by an enzyme-linked immunosorbent assay (ELISA) (ERCC1 ELISA kit, Xinfan Biosystems, Shanghai, China) and then quantified it using a standard curve with the detection range of 30–1200 pg/ml.

### Statistical analyses

The differences in clinical characteristics between cases and controls were analyzed by the Pearson chi-square test (categorical variables) and the Student’s t-test (continuous variables). The Pearson chi-square test was also used to assess Hardy-Weinberg equilibrium (HWE) of each SNP in three independent study populations. Odds ratio (OR) and their 95% confidence intervals (CIs) were calculated by logistic regression analyses to estimate the association of ERCC1 SNPs with CAD risk before or after adjusting for age, sex, BMI, smoking, alcohol drinking, hypertension, T2DM and hyperlipidemia. Genetic and environmental interactions of ERCC1 SNPs and selected stratification variables on CAD risk were evaluated by the multiplicative likelihood ratio test. When assessing severity of coronary artery atherosclerosis by Gensini score, Mann-Whitney U test and logistic regression analyses were used to analyze the association of ERCC1 SNPs with Gensini score. All the analyses above were carried out using SPSS 22.0 (SPSS Inc., Chicago, IL, USA) and P values were considered statistically significant below the cut-off value of 0.05. Also, Bonferroni correction was performed for multiple comparisons in the entire analyses. Moreover, power analyses were performed using Power and Sample Size program (PS) 3.0 (Vanderbilt University, Nashville, TN, USA).

### Data Availability

All data generated or analyzed during this study are included in this published article (and its Supplementary Information files).

## Electronic supplementary material

Supplementary Information

## References

[1] Lanktree MB, Hegele RA (2009). Gene-gene and gene-environment interactions: new insights into the prevention, detection and management of coronary artery disease. Genome Med..

[2] Willer CJ (2008). Newly identified loci that influence lipid concentrations and risk of coronary artery disease. Nat Genet..

[3] Hansson GK (2005). Inflammation, atherosclerosis, and coronary artery disease. N Engl J Med..

[4] Bennett MR (2001). Reactive oxygen species and death: oxidative DNA damage in atherosclerosis. Circ Res..

[5] Nair J, De Flora S, Izzotti A, Bartsch H (2007). Lipid peroxidation-derived etheno-DNA adducts in human atherosclerotic lesions. Mutat Res..

[6] Uryga A, Gray K, Bennett M (2016). DNA Damage and Repair in Vascular Disease. Annual review of physiology..

[7] Mahmoudi M, Mercer J, Bennett M (2006). DNA damage and repair in atherosclerosis. Cardiovasc Res..

[8] Rodier F (2009). Persistent DNA damage signalling triggers senescence-associated inflammatory cytokine secretion. Nature cell biology..

[9] de Laat WL, Jaspers NG, Hoeijmakers JH (1999). Molecular mechanism of nucleotide excision repair. Genes Dev..

[10] Sancar A, Reardon JT (2004). Nucleotide excision repair in E. coli and man. Adv Protein Chem..

[11] Ferry KV, Hamilton TC, Johnson SW (2000). Increased nucleotide excision repair in cisplatin-resistant ovarian cancer cells: role of ERCC1-XPF. Biochem Pharmacol..

[12] He KY (2009). Excision repair cross-complementing 1 expression protects against ischemic injury following middle cerebral artery occlusion in the rat brain. Gene therapy..

[13] Durik M (2012). Nucleotide excision DNA repair is associated with age-related vascular dysfunction. Circulation..

[14] Gregg SQ (2012). A mouse model of accelerated liver aging caused by a defect in DNA repair. Hepatology..

[15] Smith SC, Robinson AR, Niedernhofer LJ (2012). & Hetman, M. Downregulation of cholesterol biosynthesis genes in the forebrain of ERCC1-deficient mice. Neurobiol Dis..

[16] Viguier J (2005). ERCC1 codon 118 polymorphism is a predictive factor for the tumor response to oxaliplatin/5-fluorouracil combination chemotherapy in patients with advanced colorectal cancer. Clinical cancer research: an official journal of the American Association for Cancer Research..

[17] Chen P (2000). Association of an ERCC1 polymorphism with adult-onset glioma. Cancer epidemiology, biomarkers & prevention: a publication of the American Association for Cancer Research, cosponsored by the American Society of Preventive Oncology..

[18] Lee SY (2015). Functional intronic ERCC1 polymorphism from regulomeDB can predict survival in lung cancer after surgery. Oncotarget..

[19] Melton DW (1998). Cells from ERCC1-deficient mice show increased genome instability and a reduced frequency of S-phase-dependent illegitimate chromosome exchange but a normal frequency of homologous recombination. J Cell Sci..

[20] Yu JJ (2000). Comparison of two human ovarian carcinoma cell lines (A2780/CP70 and MCAS) that are equally resistant to platinum, but differ at codon 118 of the ERCC1 gene. Int J Oncol..

[21] Lathe R (1985). Synthetic oligonucleotide probes deduced from amino acid sequence data. Theoretical and practical considerations. J Mol Biol..

[22] Yu JJ (1997). A nucleotide polymorphism in ERCC1 in human ovarian cancer cell lines and tumor tissues. Mutat Res..

[23] Frietze S, Lan X, Jin VX, Farnham PJ (2010). Genomic targets of the KRAB and SCAN domain-containing zinc finger protein 263. J Biol Chem..

[24] Botto N (2001). Evidence for DNA damage in patients with coronary artery disease. Mutat Res..

[25] Martinet W, Knaapen MW, De Meyer GR, Herman AG, Kockx MM (2002). Elevated levels of oxidative DNA damage and DNA repair enzymes in human atherosclerotic plaques. Circulation..

[26] Carnevale R, Nocella C (2012). Alcohol and cardiovascular disease: still unresolved underlying mechanisms. Vascul Pharmacol..

[27] Li X (2014). Association of single nucleotide polymorphisms of nucleotide excision repair genes with laryngeal cancer risk and interaction with cigarette smoking and alcohol drinking. Tumour Biol..

[28] Lee-Kwon W, Park D, Bernier M (1998). Involvement of the Ras/extracellular signal-regulated kinase signalling pathway in the regulation of ERCC-1 mRNA levels by insulin. Biochem J..

[29] Prasher JM (2005). Reduced hematopoietic reserves in DNA interstrand crosslink repair-deficient Ercc1−/− mice. Embo j..

[30] Torpy JM, Burke AE, Glass RM (2009). JAMA patient page. Coronary heart disease risk factors. Jama..

[31] Ko DT (2014). Traditional cardiovascular risk factors and the presence of obstructive coronary artery disease in men and women. Can J Cardiol..

[32] Abecasis GR (2010). A map of human genome variation from population-scale sequencing. Nature..

[33] Xiao S (2015). Functional marker detection and analysis on a comprehensive transcriptome of large yellow croaker by next generation sequencing. PLoS One..

[34] Wang Z (2015). Genetic diversity and population structure of six Chinese indigenous pig breeds in the Taihu Lake region revealed by sequencing data. Anim Genet..

[35] Montorsi P (2006). Association between erectile dysfunction and coronary artery disease. Role of coronary clinical presentation and extent of coronary vessels involvement: the COBRA trial. European heart journal..

[36] Gensini GG (1983). A more meaningful scoring system for determining the severity of coronary heart disease. The American journal of cardiology..

[37] Zhang H, Bonney G (2000). Use of classification trees for association studies. Genet Epidemiol..

[38] Gu D (2006). Association study with 33 single-nucleotide polymorphisms in 11 candidate genes for hypertension in Chinese. Hypertension..

