# Supplementary material for: Polymorphism in ERCC1 confers susceptibility of coronary artery disease and severity of coronary artery atherosclerosis in a Chinese Han population
Source: Sci Rep. 2017 Jul 25;7:6407. doi: 10.1038/s41598-017-06732-9 (PMC5526898; doi:10.1038/s41598-017-06732-9)
Supplement: Supplementary file 1 — Supplementary Information [file 41598_2017_6732_MOESM1_ESM.pdf]

Polymorphism in ERCC1 confers susceptibility of coronary artery disease and severity of coronary artery atherosclerosis in a Chinese Han population

Shuai Zhang[1], Xue-bin Wang[1], Ya-di Han[1], Chen-ling Xiong[1], Ye Zhou[1], Chen Wang[1], Ze-jin Liu[2], Na Yang[1], Fang Zheng[1 *]

1 Center for Gene Diagnosis, Zhongnan Hospital of Wuhan University, Wuhan, Hubei, China.

2 Center of Clinical Laboratory, Wuhan Asia Heart Hospital, Wuhan, Hubei, China

* Corresponding author: Fang Zheng, Center for Gene Diagnosis, Zhongnan Hospital of Wuhan University, Donghu Road 169#, Wuchang District, Wuhan 430071, Hubei, China

Email address: zhengfang@whu.edu.cn

Telephone number: 862767813233

Fax number: 86-2767811832

Supplementary materials and methods

Supplementary genotyping methods

Genomic DNA samples were fragmented and then ligated with a unique barcode using polymerase chain reaction (PCR). Short fragments were purified with Omega Gel Extraction Kit (Step 1 in supplementary Fig. S1) and resolved with NEBNext End prep kit for ending reparation and poly(A) addition (Step 2 in supplementary Fig. S1). After connecting to adaptors for using NEBNext Library Prep Kit for illumina and subsequent purification (Step 3 in supplementary Fig. S1), DNA fragments were used as templates for the following PCR amplification (Step 4 in supplementary Fig. S1). Next, according to DNA concentration, only samples meeting the requirement of quality detection were pooled to construct a sequencing library (Step 5 in supplementary Fig. S1). Finally, the sequencing libraries were sequenced on an Illumina Miseq system with a paired-end pattern and the data were stored in FASTQ format. The short reads with high quality, which withstood next generation sequencing quality control (NGSQC)[1], were used for further downstream sequence analyses. According to specific barcode of each DNA samples and the known location of SNPs, we could know the proportion of each bases at the SNP locus in every DNA samples. Genotypes were determined by Bayesian formula based on the above proportion and sequencing depth, mentioned in previous article[2].

Definition of clinical characteristics

Individuals who smoked ≥ 100 cigarettes in their lifetime were defined as "smokers", which included ever smokers and current smokers. An ever smoker was a person who had quit smoking at least 1 year prior to interview. Subjects with alcohol consumption at least once a week for ≥ 1 year were defined as "alcohol drinkers". One drink was defined as 375 ml of beer (13.6 g of ethanol), 118 ml of wine (11.7 g of ethanol), or 30 ml of western or Chinese hard liquor (10.9 g of ethanol). Hypertension

was defined as ongoing therapy for hypertension, systolic blood pressure (SBP) of ≥ 140mmHg or diastolic blood pressure (DBP) of ≥ 90mmHg. Type 2 diabetes mellitus (T2DM) was defined as ongoing therapy for diabetes or fasting plasma glucose (FPG) levels of ≥ 7.0 mmol/L, or with plasma glucose levels of ≥ 11.1mmol/L, or a 2-h plasma glucose level of ≥ 11.1mmol/L during an oral glucose tolerance test. Hyperlipidemia was defined as hypercholesterolemia (serum TC > 5.72 mmol/L), high levels of LDL-c (> 3.1 mmol/L), low levels of HDL-c (< 0.9 mmol/L), hypertriglyceridemia (serum TG > 1.70 mmol/L).

References

1 Patel, R. K. & Jain, M. NGS QC Toolkit: a toolkit for quality control of next generation sequencing data. *PloS one* **7**, e30619 (2012).

2 Li, H., Ruan, J. & Durbin, R. Mapping short DNA sequencing reads and calling variants using mapping quality scores. *Genome research* **18**, 1851-1858 (2008).

Supplementary Tables

Table S1. Characteristics of 3 SNPs in ERCC1 gene.

SNP	Position *	Location	Major/Minor allele	MAF (%) †			P (HWE) ‡		
				Study 1	Study 2	Study 3	Study 1	Study 2	Study 3
rs3212986	Chr19: 45409478	3'-UTR	G/T	31.6	33.9	—	0.380	0.580	—
rs11615	Chr19: 45420395	Exon Ⅳ	C/T	21.8	22.8	21.8	0.562	0.381	0.122
rs2298881	Chr19: 45423658	Intron Ⅰ	C/A	38.9	38.0	—	0.937	0.321	—

Chr, chromosome; UTR, untranslated region; MAF, minor allele frequency; HWE, Hardy-Weinberg equilibrium.

* Information for chromosome position was based on NCBI genome build 38.2.

† MAF was calculated from the genotype data in our controls.

‡ The P value for HWE was calculated from the genotype data in our controls.

Table S2. Association of ERCC1 SNPs with CAD risk in three independent study populations

SNPs	Discovery set (Study 1)				Validation set (Study 2)				Replication set (Study 3)			
	CAD N (%)	Control N (%)	OR(95%CI) *	P*	CAD N (%)	Control N (%)	OR(95%CI) *	P*	CAD N (%)	Control N (%)	OR(95%CI) *	P*
rs11615												
C	1202(74.5)	1276(78.2)	1(Ref)		1665(74.1)	1726(77.2)	1(Ref)		1639(74.0)	1671(78.2)	1(Ref)	
T	410(25.5)	356(21.8)	**1.27(1.07-1.50)**	**0.006**	583(25.9)	510(22.8)	**1.19(1.03-1.38)**	**0.021**	575(26.0)	465(21.8)	**1.23(1.05-1.43)**	**0.01**
CC	449(55.7)	496(60.8)	1(Ref)		615(54.7)	661(59.1)	1(Ref)		602(54.4)	645(60.4)	1(Ref)	
CT	304(37.7)	284(34.8)	1.21(0.97-1.49)	0.089	435(38.7)	404(36.1)	1.16(0.96-1.40)	0.127	435(39.3)	381(35.7)	1.21(0.99-1.47)	0.054
TT	53(6.6)	36(4.4)	**1.81(1.15-2.86)**	**0.011**	74(6.6)	53(4.7)	**1.53(1.02-2.30)**	**0.038**	70(6.3)	42(3.9)	**1.61(1.04-2.54)**	**0.033**
TT+CT	357(44.3)	320(39.2)	**1.27(1.04-1.56)**	**0.022**	509(45.3)	457(40.9)	**1.20(1.01-1.44)**	**0.048**	485(43.8)	423(39.6)	**1.25(1.04-1.51)**	**0.019**
rs2298881												
C	1016(63.0)	997(61.1)	1(Ref)		1395(62.1)	1387(62.0)	1(Ref)					
A	596(37.0)	635(38.9)	0.90(0.78-1.05)	0.177	853(37.9)	849(38.0)	1.01(0.89-1.15)	0.843				
CC	322(40.0)	304(37.2)	1(Ref)		431(38.4)	438(39.2)	1(Ref)			NA		
AC	372(46.1)	389(47.7)	0.88(0.71-1.10)	0.271	533(47.4)	511(45.7)	1.02(0.84-1.24)	0.84				
AA	112(13.9)	123(15.1)	0.83(0.61-1.13)	0.236	160(14.2)	169(15.1)	1.02(0.78-1.34)	0.88				
AA+AC	484(60.0)	512(62.8)	0.87(0.71-1.07)	0.19	693(61.6)	680(60.8)	1.02(0.85-1.23)	0.83				
rs3212986												
G	1118(69.4)	1116(68.4)	1(Ref)		1529(68.0)	1479(66.1)	1(Ref)					
T	494(30.6)	516(31.6)	0.96(0.82-1.12)	0.622	719(32.0)	757(33.9)	0.92(0.80-1.05)	0.208				
GG	379(47.0)	387(47.4)	1(Ref)		531(47.2)	485(43.4)	1(Ref)			NA		
GT	360(44.7)	342(41.9)	1.09(0.88-1.35)	0.411	467(41.6)	509(45.5)	0.85(0.70-1.03)	0.094				
TT	67(8.3)	87(10.7)	0.79(0.55-1.13)	0.194	126(11.2)	124(11.1)	0.91(0.67-1.23)	0.535				
TT+GT	427(53.0)	429(52.6)	1.03(0.84-1.26)	0.767	593(52.8)	633(56.6)	0.86(0.72-1.03)	0.106				

CAD, coronary artery disease; N, number; OR (95% CI), odds ratio (95% confidence interval); Ref, reference; NA, not available.

* P value from logistic regression after adjustment for age, sex, BMI, smoking status, alcohol drinking and histories of hypertension, hyperlipidemia and T2DM.

Bold values are statistically significant with $P < 0.05$.

Table S3. Primer details and PCR conditions for library preparation, sequencing and RT-qPCR analyses in our study.

Method	Variable	Primers	Length (bp)	Temp (℃)
Adding barcode *	Rs11615	Forward: GACCAGCTCGACCTGCCGCTCCCTATTGATGGCTTCTGC	251	56
		Reverse: TCCTCGCCCTTGCTCACCAGCCTGAAGTCTGGGGTGG		
	Rs2298881	Forward: GACCAGCTCGACCTGCCGCTCAGGTCCACAAGTCCCAT	241	56
		Reverse: TCCTCGCCCTTGCTCACCAGCCAATCATTGCCGAGTCT		
	Rs3212986	Forward: GACCAGCTCGACCTGCCGCTAATAAATCGTCCTCCCAG	260	56
		Reverse: TCCTCGCCCTTGCTCACCAGGGGACAAGAAGCGGAAG		
PCR amplification †		Index primer: ATCACGAC	—	65
		Universal primer: AATGATACGGCGACCACCGAGATCTACACTCTTC CCTACACGACGCTCTTCCGATCT		
qPCR ‡		Forward: AATGATACGGCGACCACCGAGAT	—	60
		Reverse: CAAGCAGAAGACGGCATACGA		
RT-qPCR §	ERCC1	Forward: CTATGAGCAGAAACCAGCG	143	57
		Reverse: GTTCCAGAGATCCAAATGTG		
	GAPDH	Forward: GAAGGTGAAGGTCGGAGTC	226	57
		Reverse: GAAGATGGTGATGGGATTTC		
Sequencing **	Rs11615	Forward: CCCTATTGATGGCTTCTGC	171	58
		Reverse: CCTGAAGTCTGGGGTGG		
	Rs2298881	Forward: CAGGTCCACAAGTCCCAT	161	58
		Reverse: CCAATCATTGCCGAGTCT		
	Rs3212986	Forward: TGAAAGTACCCTGATGACCC	308	58
		Reverse: GATGCCAGAGACAGTGCC		

Temp., annealing temperature; qPCR, quantitative PCR; RT-qPCR, real time-quantitative PCR.

* PCR reaction for step 1 was performed in a volume of 20 μL containing 25 ng of genomic DNA, 0.5 μL of each primer (1 mM), 0.5 μL of barcode F/R (10mM), 10 μL of 2* PCR Mix. The cycling conditions are 94 ℃ for 3 min followed by 35 cycles of 30 s at 94 ℃, 30 s at 56 ℃ and 30 s at 72 ℃, and final extensions of 5 min at 72 ℃ and then hold on 4 ℃.

† PCR reaction for step 4 was performed in a volume of 50 μL containing 23 μL of DNA, 25 μL of NEB Next High Fidelity 2*PCR Mix , 1 μL of index/universal primer. The cycling conditions are 98 ℃ for 30 s followed by 6—15 cycles of 10 s at 98 ℃, 30 s at 65 ℃ and 30 s at 72 ℃, and final extensions of 5 min at 72 ℃ and then hold on 4 ℃.

‡ qPCR analysis in step 5 was performed in a final volume of 20 μL reaction mixture containing 10 μL of SYBR Green Master mix (Bio-rad), 5 pmol of each primers and 50 ng of cDNA products. The cycling conditions are 95 ℃ for 2 min and 40 cycles of 95 ℃ for 15 s, 60 ℃ for 15 s and 72 ℃ elongation for 45s.

§ RT-qPCR analysis was performed for detecting ERCC1 mRNA expression in a final volume of 20 μL reaction mixture containing 10 μL of 1 X SYBR Green Master mix (Bio-rad), 5 pmol of each primer and 50 ng of cDNA products. The cycling conditions for RT-qPCR are 95 ℃ for 5 min and 40 cycles of 95 ℃ for 30 s and 57 ℃ for 30s and 72 ℃ elongation for 30s.

**The cycling conditions for sequencing are 95 ℃ for 5 min, 35 cycles of 30 s at 95 ℃, 30s at 58 ℃ and 45 s at 72 ℃, and final extensions of 5 min at 72 ℃.

Supplementary Figures

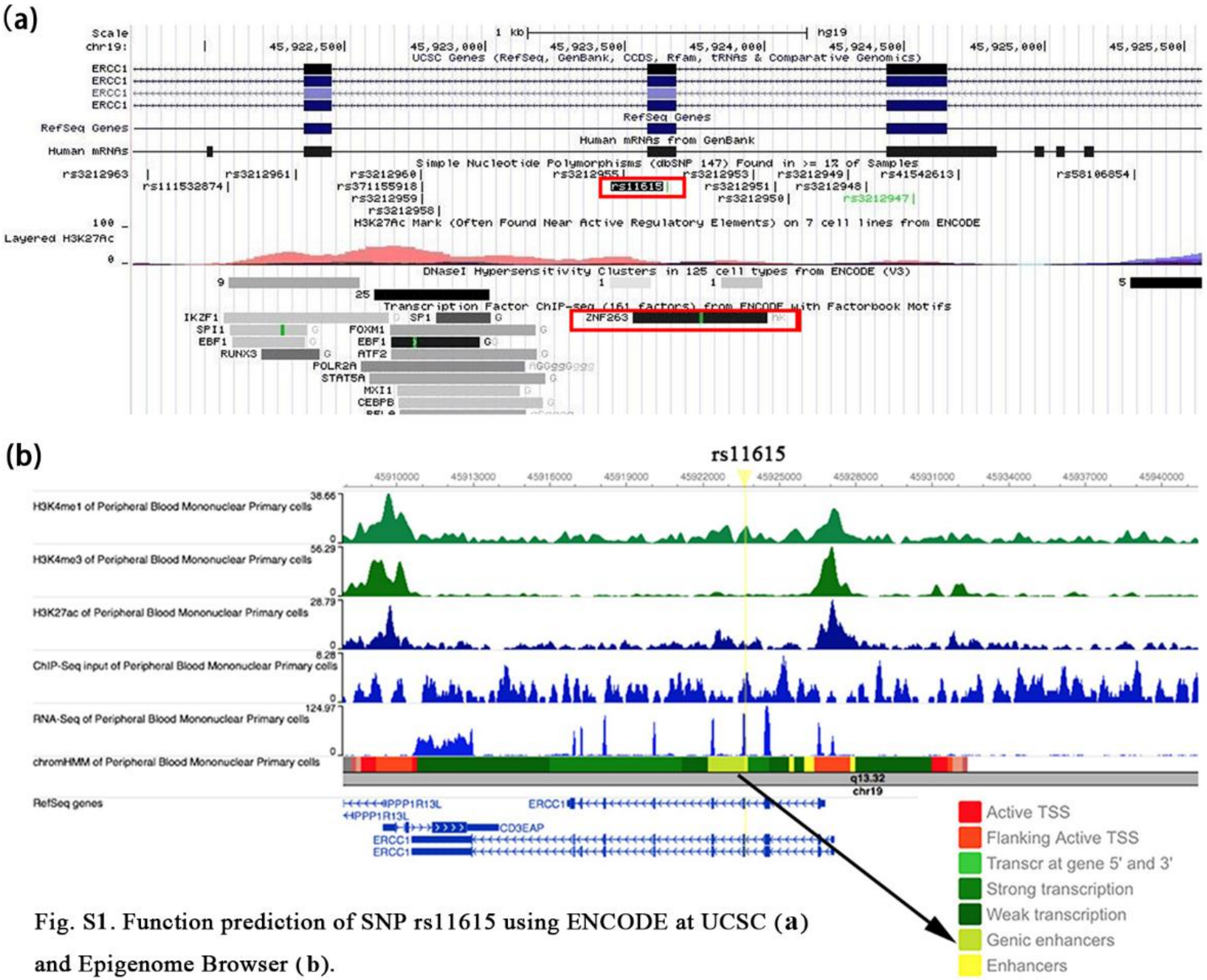

Fig. S1. Function prediction of SNP rs11615 using ENCODE at UCSC (a) and Epigenome Browser (b).

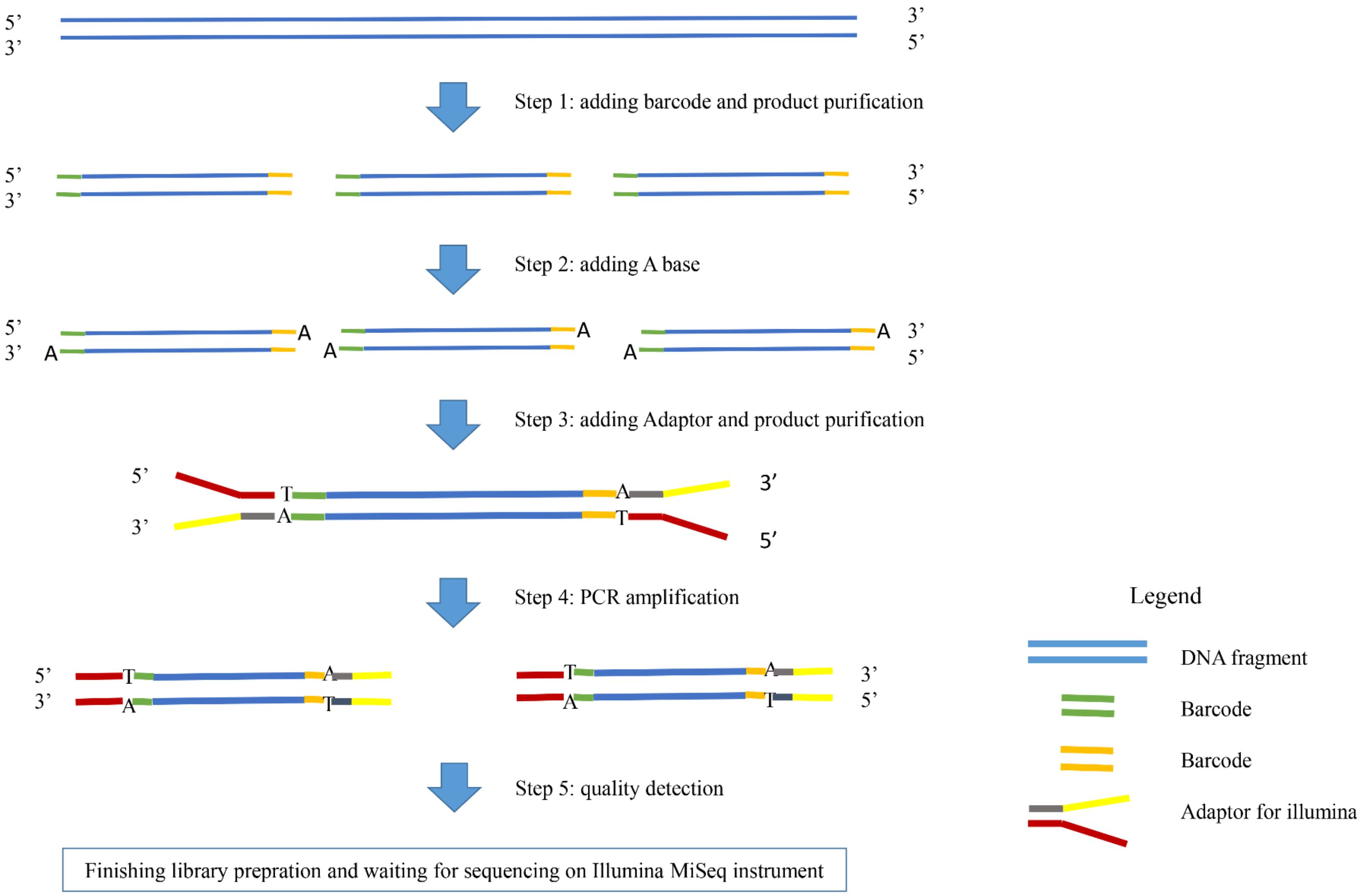

Fig. S2. Flow chart of high-throughput sequencing using illumina Miseq system. Operation of each step was described in supplementary genotyping methods.

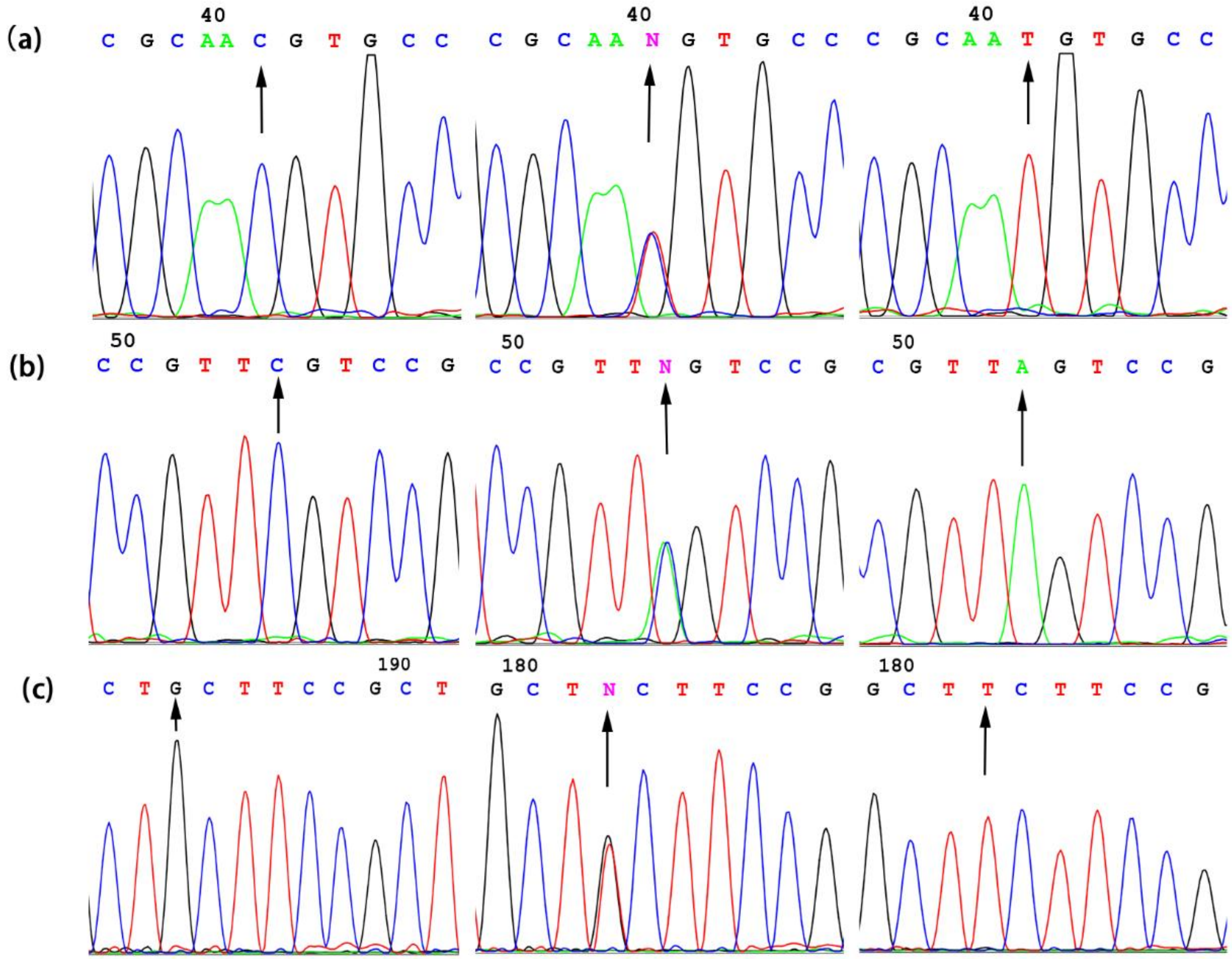

Fig. S3. Direct sequencing analyses for different genotypes of three SNPs. The three genotypes of SNPs rs11615 (C>T), rs2298881 (C>A) and rs3212986 (G>T) are shown in a, b, and c respectively.
